# Pharmacokinetics of Selected Anticancer Drugs in Elderly Cancer Patients: Focus on Breast Cancer

**DOI:** 10.3390/cancers8010006

**Published:** 2016-01-02

**Authors:** Marie-Rose B.S. Crombag, Markus Joerger, Beat Thürlimann, Jan H.M. Schellens, Jos H. Beijnen, Alwin D.R. Huitema

**Affiliations:** 1 Department of Pharmacy & Pharmacology, The Netherlands Cancer Institute/Slotervaart Hospital, Amsterdam 1066 CX, The Netherlands; marie-rose.crombag@slz.nl (M.-R.B.S.C.); jos.beijnen@slz.nl (J.H.B.); 2 Department of Clinical Pharmacology, The Netherlands Cancer Institute, Amsterdam 1066 CX, The Netherlands; j.schellens@nki.nl; 3 Department of Medical Oncology, Cantonal Hospital, St. Gallen 9007, Switzerland; 4 Breast Center, Cantonal Hospital, St. Gallen 9007, Switzerland; beat.thuerlimann@kssg.ch; 5 Department of Pharmaceutical Sciences, Division of Pharmacoepidemiology & Clinical Pharmacology, Utrecht University, Utrecht 3508 TC, The Netherlands; 6 Department of Medical Oncology, The Netherlands Cancer Institute, Amsterdam 1066 CX, The Netherlands

**Keywords:** pharmacology, pharmacokinetics, anticancer drugs, elderly, breast cancer

## Abstract

Background: Elderly patients receiving anticancer drugs may have an increased risk to develop treatment-related toxicities compared to their younger peers. However, a potential pharmacokinetic (PK) basis for this increased risk has not consistently been established yet. Therefore, the objective of this study was to systematically review the influence of age on the PK of anticancer agents frequently administered to elderly breast cancer patients. Methods: A literature search was performed using the PubMed electronic database, Summary of Product Characteristics (SmPC) and available drug approval reviews, as published by EMA and FDA. Publications that describe age-related PK profiles of selected anticancer drugs against breast cancer, excluding endocrine compounds, were selected and included. Results: This review presents an overview of the available data that describe the influence of increasing age on the PK of selected anticancer drugs used for the treatment of breast cancer. Conclusions: Selected published data revealed differences in the effect and magnitude of increasing age on the PK of several anticancer drugs. There may be clinically-relevant, age-related PK differences for anthracyclines and platina agents. In the majority of cases, age is not a good surrogate marker for anticancer drug PK, and the physiological state of the individual patient may better be approached by looking at organ function, Charlson Comorbidity Score or geriatric functional assessment.

## 1. Introduction

The majority of patients diagnosed with cancer are aged 65 years and older, and it is predicted that in the period from 2010–2030, the cancer incidence in the elderly will increase by 67% [1]. Elderly patients (often defined as ≥65 years of age) are routinely treated with anticancer drugs; however, the pharmacokinetics and pharmacodynamics (PK and PD) of these agents have not been thoroughly investigated in this population thus far. Despite the increasing size of the geriatric population and specific guidance on studies in geriatrics [2], the elderly patient is mostly excluded from clinical trials due to short life expectancy, performance status, comorbidities and/or comedication [3,4]. In clinical trials, the underrepresentation of older patients has been substantial, even in cancer types that predominantly occur in the elderly [5,6,7]. FDA and EMA consider dose- and concentration-response information to be key for determining the safety and effectiveness of drugs [8,9]. Identification of the appropriate dosage regimen of an anticancer drug for the respective target population, including elderly patients, is crucial to ensure its safety and effectiveness in clinical practice.

In daily practice, elderly breast cancer patients have a higher risk of death from breast cancer compared to their younger peers [10]. This may be ascribed to a combination of factors, including tumor biology, patient characteristics and deviating treatment regimens applied in this population. Treatment strategies in elderly patients are often less stringent, and there is a general risk of undertreatment in elderly patients [10]. Elderly patients receiving anticancer drugs may have an increased risk to develop treatment-related toxicities compared to their younger peers. However, a potential PK basis for this increased risk has not consistently been established yet. Therefore, this review was designed to: (1) briefly outline the influence of aging on selected anticancer drug PK; and (2) describe the PK basis for potential dose adjustments of anticancer agents frequently administered to elderly breast cancer patients.

## 2. Influence of Aging on Drug Pharmacokinetics

With increasing age, multiple physiological parameters alter, which may substantially influence the PK of anticancer drugs. In elderly patients, the PK profile can be influenced by altered distribution, metabolism and elimination parameters, while changes in absorption rarely led to clinically-relevant differences, as depicted in Table 1 [11,12,13,14]. Changes in gastric pH may have variable impacts on anticancer drug absorption, while absorption of Class II oral therapeutic drugs, including tyrosine kinase inhibitors and endocrine agents, increases with increasing gastric pH. Another typical example includes capecitabine, with a higher absorption in elderly patients with a higher gastric pH, similar to increased absorption in the fed compared to the fasted state.

**Table 1:** Influence of aging on pharmacokinetics (PK) parameters.

	Parameter	Direction	Effect on Exposure
**Absorption [11,13]**	Gastrointestinal pH	↑	↓↑
	Gastric emptying time	↑	↓
	Motility	↓	↓
	Splanchnic blood flow	↓	↓
	Absorptive surface	↓	↓
**Distribution [11,13]**	Body composition		
	*Body fat*	↑	↑ *
	*Plasma volume*	↓	↑** §**
	*Total body water*	↓	↑ **§**
	*Intra-/extracellular body fluid*	↓	↑ **§**
	Plasma proteins		
	*Serum albumin*	↓	↑ FF
	*Bilirubin*	↓	↑ FF
	*Erythrocytes*	↓	↑ FF
	*Serum α1-acid glycoprotein*	↑	↓ FF
**Metabolism [12]**	Hepatic blood flow	↓	↑
	Hepatic mass	↓	↑
	CYP P450 enzymes	0/↓	(↑)
**Elimination [11,13]**	Renal blood flow/glomerular filtration	↓	↑
	Tubular secretion	↓	↑

* For lipophilic drugs; § for hydrophilic drugs; FF = free fraction.

These multifactorial and complex changes make it difficult to predict the net effect of aging on the PK profile of a specific drug administered to elderly breast cancer patients. Besides these physiological changes, multiple other factors contribute to the complexity of anticancer drug treatment in the elderly patient. Firstly, elderly patients often have several comorbidities and receive comedication that may negatively affect anticancer treatment [12,15,16]. For instance, patients with diabetes mellitus encountered more chemotherapy-related toxicities when receiving adjuvant chemotherapy for breast cancer compared to the non-diabetic control group [17]. While the exact reasons for this effect are unclear, a higher fat proportion in the elderly patient may result in impaired anticancer drug disposition and increased toxicity from various chemotherapy regimens. Furthermore, comorbidities were determined to significantly influence mortality rates in elderly patients diagnosed with cancer, regardless of the stage of disease at diagnosis [18,19].

Frailty, decreased mobility and impaired performance status may come with aging, although there is a large interindividual variability in the extent and rate of these processes. These factors are important parameters that help differentiate between geriatric and non-geriatric patients. Because the proportion and absolute number of elderly cancer patients is steadily increasing, and with it the detrimental impact of suboptimal cancer treatment, it is important to have adequate evidence-based anticancer drug regimens that are tailored to use in elderly breast cancer patients.

## 3. Methodology

A literature search was performed using the PubMed electronic database to identify available PK data on anticancer drugs that were frequently administered to elderly breast cancer patients, excluding endocrine compounds. The following keywords were used: pharmaco-kinetic; kinetic; PK; AUC; concentration; Cmax; half-life; in conjunction with aged, elderly, geriatric, ageing, aging, older, old people, frail elderly; which was subsequently combined with each anticancer agent included in this review, both by its generic and brand name. Results were limited to publications in humans, written in English, which were published through January 2015. Furthermore, the Summary of Product Characteristics (SmPC) and available drug approval reviews, as published by EMA and FDA, were analyzed to evaluate additional PK information. Publications that describe age-related PK profiles of all anticancer drugs included in this review were selected and included. Cited references were hand-searched and included when the set inclusion criteria were met.

## 4. Results

Table 2 depicts all studies investigating the influence of increasing age on the PK of anticancer drugs included in this review.

### 4.1. Taxanes

#### 4.1.1. Docetaxel

Docetaxel is a highly lipophilic taxane anticancer drug that is >94% bound to plasma proteins [20,21]. Altered distribution of docetaxel can be expected in the elderly patient who has an altered body composition and increased serum α1-acid glycoprotein [11,12]. With only a small fraction excreted renally, docetaxel undergoes hepatic metabolism by CYP3A4 into inactive metabolites and mainly undergoes biliary excretion [21,22]. The activity of CYP3A4 may be preserved with aging, although there is some controversy on this topic [11,23,24]. The number of older patients included in several trials was insufficient to determine differences in PK and PD related to age. For this reason, no evaluation of pivotal trial data has been conducted for elderly breast cancer patients [20]. Four different studies found no significant PK differences in elderly patients (*n* = 50, *n* = 54, *n* = 40 and *n* = 19, respectively) compared to younger patients [25,26,27,28]. It should be noted that the latter study lacked a control group, including only patients aged 65 years or older [28]. On the contrary, two other studies (*n* = 29 and *n* = 20, respectively) documented a significant decrease in docetaxel clearance with increasing age [29,30]. A subsequent larger study (*n* = 601) supported this significant effect of aging on docetaxel PK, although the observed decrease in clearance of 7% is not usually seen as clinically relevant [31]. Although apparently not PK-driven, a significant increase in (hemato-)toxicity was observed in various studies [25,27,28,31], supporting FDA’s statement for cautious dose selection in elderly patients [20]. Nonetheless, in EMA’s SmPC, it was stated that, based on population PK analysis [32], dose reductions in elderly patients are not recommended [33].

**Table 2:** Overview of studies assessing the pharmacokinetics of anticancer drugs in elderly patients.

Author	Year	Treatment	Dose	Indication	N	Age (Mean, Range)	Patient Groups	Outcome (Elderly *vs.* Non-Elderly)	Comments
**Yamamoto**	2000	Docetaxel	60 mg/m^2^	NSCLC	29	58 (32–76)	NA	SIG	
**Bruno**	2001	Docetaxel	75–100 mg/m^2^	Solid tumors	601	56 (38–71) ^§^	NA	SIG	Estimated decrease in CL at age 71 years was 7%; reported to be not clinically relevant
**Minami**	2004	Docetaxel w/cisplatin	Docetaxel:	NSCLC	50	76 (75–86)	Docetaxel:	Docetaxel:	Elderly received a lower doxorubicin dose per protocol, resulting in a significantly lower AUC, with no difference in CL.
							≥75 y; *n* = 25	CL: −0.7%	
			≥75 y: 20 mg/m^2^					Vd: −22%	
			<75 y: 35 mg/m^2^					AUC: −44% *	
			Cisplatin: 25 mg/m^2^				<75 y; *n* = 25		
						56 (39–73)	Cisplatin:	Cisplatin:	
							≥75 y; *n* = 24	CL: −6%	
								Vd: +7%	
								AUC: +3%	
							<75 y; *n* = 27		
**Slaviero**	2004	Docetaxel	40 mg/m^2^	Solid tumors	54	63 (40–83)		NS	
**Ten Tije**	2005	Docetaxel	75 mg/m^2^	Solid tumors	40	71 (65–80)	≥65 y; *n* = 20	Cmax: −15%	
								AUC: +6%	
						53 (26–64)	<65 y; *n* = 20	Vdss: +15%	
								T½: +13%	
**Hurria**	2006	Docetaxel	35 mg/m^2^	Solid tumors	19	75 (66–84)	>65 y; *n* = 19	NS	No control group
**Michael**	2012	Docetaxel	35–75 mg/m^2^	MC, NSCLC	20	62 (41–77)	NA	SIG	
**Borkowski**	1994	Paclitaxel	90–265 mg/m^2^	Solid tumors	16	53 (38–72)	NA	NS	
**Huizing**	1997	Paclitaxel–carboplatin	Paclitaxel: 100–250 mg/m^2^	NSCLC	55	56 (38–74)	NA	Paclitaxel and carboplatin: SIG	Exclusion of patients aged ≥75 y
			Carboplatin: 300–400 mg/m^2^						
**Nakamura**	2000	Paclitaxel	210 mg/m^2^	NSCLC	14	NA	≥70 y; *n* = 3	Cmax: −8%	
								AUC: −0.5%	
								MRT: +8%	
							<70 y; *n* = 11	T ½: +14%	
								CL: +4%	
								D > 0.1 μM: +0.9%	
**Fidias**	2001	Paclitaxel	90 mg/m^2^	NSCLC	13	76 (70–85)	≥70 y; *n* = 13	NS	No control group
**Smorenburg**	2003	Paclitaxel	≥70 y: 80 mg/m^2^	MC	23	NA (22–84)	≥70 y; *n* = 8	Unbound paclitaxel:	
								Cmax: +40%	
			<70 y: 100 mg/m^2^					AUC: +49%	
							<70 y; *n* = 15	CL: −50% *	
								Vss: −57% *	
								T ½: −17%	

								Total paclitaxel:	
								Cmax: −5%	
								AUC: +16%	
								CL: −20% *	
**Lichtman**	2006	Paclitaxel	175 mg/m^2^	Non-hematological malignancies	122	NA (55–86)	55–64 y; *n* = 46	≥65 y:	
							65–74 y; *n* = 44	AUC: +29%	
								CL: −21%	
							75–86 y; *n* = 32		
								≥75 y:	
								AUC: +30%	
								CL: −19%	
**Joerger**	2006	Paclitaxel	100–250 mg/m^2^	Solid tumors	168	56 (33–86)	NA	VMel: −5% per 10-y increase in age *	
**Joerger**	2012	Paclitaxel	76–311 mg/m^2^	Solid tumors	273	56 (33–75)	≥70 y; *n* = 19	VMel: −13% per 10-y increase in age *	
							<70 y; *n* = 254		
**Robert**	1983	Doxorubicin	12.5–50 mg/m^2^	MC, lymphoma, others	37	51 (17–74)	NA	≥70 y:	Also reported that doxorubicin CL of the distribution phase was significantly influenced by aging in 26 patients studied by Piazza *et al.*
								CL of distribution phase: −30% *	
								Total CL: −23%	
**Dobbs**	1995	Doxorubicin	<25 m–100 mg/m^2^	MC, lymphoma, others	27	54 (27–75)	NA	NS	
**Dees**	2000	Doxorubicin–cyclophosphamide	Doxorubicin: 60 mg/m^2^	MC	14	NA (35–79)	≥65 y; *n* = 7	NS	Increasing age (continuous) was weakly correlated with Vdss, but not with CL; no significance was reached with age as a categorical variable
			Cyclophosphamide: 600 mg/m^2^						
							<65 y; *n* = 7		
**Li**	2003	Doxorubicin	30–75 mg/m^2^	MC, lymphoma, sarcoma, others	56	50 (12–74)	NA	SIG	
**Joerger**	2007	Doxorubicin–cyclophosphamide	Doxorubicin: 60 mg/m^2^	MC	65	56 (29–81)	NA	Doxorubicin: CL: −9% per 10-y increase of age *	
								Cyclophosphamide: NS	
			Cyclophosphamide: 600 mg/m^2^						
**Jakobsen**	1991	Epirubicin	40–135 mg/m^2^	MC	78	NA (31–74)	NA	NS	
**Wade**	1992	Epirubicin	25–100 mg/m^2^	MC, lymphoma, sarcoma	36	NA (20–73)	≥70 y; *n* = 1	SIG	Significant in women only, no elderly men were included.
							<70 y; *n* = 35		
									Predicted CL: −34% (70 *vs.* 25 y)
**Eksborg**	1992	Epirubicin	60 mg/m^2^	MC	66	61 (36–78)	NA	SIG	
**Sorio**	1997	Vinorelbine (iv)	30 mg/m^2^	MC	10	72 (66–81)	≥66 y; *n* = 10	NS	No control group
**Gauvin**	2000	Vinorelbine (iv)	20–30 mg/m^2^	Solid tumors	12	74 (66–79)	≥66 y; *n* = 12	SIG	No control group
**Gauvin**	2002	Vinorelbine (iv)	20–30 mg/m^2^	Solid tumors	27	NA (66–79)	≥66 y; *n* = 27	SIG	No control group
									ECOG 0–3
**Variol**	2002	Vinorelbine (iv/po)	Vinorelbine (iv): 20–45 mg/m^2^	Solid tumors	iv: 64	iv: 55 (27–72)	NA	NS	iv same as Nguyen: 3 phase I studies
			Vinorelbine (po): 60–100 mg/m^2^		po: 175	po: 57 (21–77)			
**Nguyen**	2002	Vinorelbine (iv)	20–45 mg/m^2^	Solid tumors	64	55 (27–73)	NA	NS	3 phase I studies
**Lush**	2005	Vinorelbine (iv)	30 mg/m^2^	Solid tumors	20	57 (40–74)	≥65 y; *n* = 14	Cmax: +26%	Age mean (range) for total group of 27 pts, of which 20 received iv vinorelbine
								T ½: +6%	
							<65 y; *n* = 6		
								AUC: +30%	
								CL: −17%	
**Wong**	2006	Vinorelbine (iv)	Flat dose: 60 mg	Solid tumors	34	63 (43–81)	NA	NS	Age mean (range) for total group of 43 pts, of which 34 pts were included for PK analysis
**Puozzo**	2004	Vinorelbine (po)	60 mg/m^2^	Solid tumors	48	74 (70–82)	≥70 y; *n* = 48	AUC: +11%	Phase II including elderly compared w/phase I reference population; same population as Gridelli *et al.*
							Phase I <70 y population: 56 (31–82); *n* = 52	Cmax: +10%	
								CL: −2%	
								T ½: +7%	
**Gridelli**	2006	Vinorelbine (po)	60 mg/m^2^, after 3 cycles: 80 mg/m^2^	NSCLC	48	74 (70–82) *	≥70 y; *n* = 48	NS	No control group; age mean (range) for total group of 56 patients, of which 48 pts were included for PK analysis (not mentioned in Puozzo *et al.*)
**Port**	1991	5-FU	320–960 mg/m^2^	Solid tumors	26	53 (43–75)	NA	CL: −16% (70 y *vs.* 50 y)	
**Milano**	1992	5-FU	365–1224 mg/m^2^	Squamous cell carcinoma of head and neck	360	62 (25–91)	>70 y: *n* = 58	NS	Only 5 elderly women included
							51–70 y: *n* = 245		
							≤50 y: *n* = 57		
**Denham**	1999	5-FU–cisplatin	5-FU: 800 mg/m^2^	Esophageal cancer	44	72 (42–91)	NA	5FU: SIG	
			Cisplatin: 80 mg/m^2^						
**Duffour**	2010	5-FU	NA	CRC	103	≥65 y: 70 (65–80)	≥65 y: *n* = 48	Cycle 1:	
								CL: −3%	
						<65 y: 59 (33–64)	<65 y: *n* = 55	Vd: −8%	
								T ½: −4%	
								AUC: 0.5%	
								Cycle 2:	
								AUC: 10%	
**Mueller**	2013	5-FU	400 mg/m^2^ bolus, followed by 2400 mg/m^2^ **	Solid tumors	31	63 (31–81)	≥65 y: *n* = 14	NS	
							<65 y: *n* = 17		
**Cassidy**	1999	Capecitabine	Flat dose: 2000 mg	Solid tumors	25	63 (41–80)	NA	NS	Bioequivalence study of two tablet formulations
**Louie**	2013	Capecitabine	1000 mg/m^2^	CRC	29	≥70 y: 77 ± 5	≥70 y: *n* = 24	Capecitabine:	
								Cmax: +200% *	
								T ½: +5%	
								AUC: +150% *	
								CL: −71% *	
						<60 y: 55 ± 3	<60 y: *n* = 5	Vd: −74% *	
								5-FU:	
								Cmax: −23%	
								T ½: −10%	
								AUC: −26%	
**Daher Abdi**	2014	Capecitabine	1250–2300 mg/m^2^	MC, CRC	20 + 40	≥75 y: 81 (75–92)	≥70 y: *n* = 20	NS	PK data of 40 patients <75 y from 2 previous phase I trials
						<75 y: 55 (30–73)	<75 y: *n* = 40		
**Merino-Sanjuan**	2011	Carboplatin–gemcitabine	Carboplatin:	NSCLC	24	≥70 y: 77 (71–81)	NA	Carboplatin: CL: −31% *	Age mean (range) for total group of 33 pts, of which 24 pts were included for PK analysis
			≥70 y: AUC 4 *vs.*						
						<70 y: 58 (44–66) ^§^			
			<70 y: AUC 5						
**Yamamoto**	1995	Cisplatin	80 mg/m^2^	NSCLC	23	61 (41–81)	>70 y: *n* = 8	SIG	
							≤70 y: *n* = 15		
**Gupta**	2012	Trastuzumab emtansine	1.2–4.8 mg/kg	MC	273	54 (SD 10 y)	NA	NS	87% of pts received 3.6 mg/kg
**Lu**	2014	Trastuzumab emtansine	1.2–4.8 mg/kg	MC	671	53 (27–84)	>75 y: *n* = 16	NS	95% of pts received 3.6 mg/kg
							65–75 y; *n* = 78		
							<65 y; *n* = 577		
**Motzer**	2008	Everolimus	Flat dose: 10 mg/day	RCC	272	61 (27–85)	NA	NS	As reported in the FDA’s drug approval review

* Significant effect, as defined in the original publication; ^§^ age median/mean (range) concerns total group of included patients in the study, of which only a part was included for PK analysis of the specific administrated treatment drug; ** as simulated using NONMEM, whereas the actual dosing range was not provided, yet was given as part of FOLFOX, FOLFIRI, 5-fluorouracil (5-FU) or FOLFIRINOX; NS = non-significant influence of aging on PK parameters, as displayed when the percentage of change of PK parameters could not be ascertained from the article, e.g., when results were presented using population PK modeling; *N* = patient number; SIG = significant influence of aging on PK parameters, as displayed when the percentage of change of PK parameters could not be ascertained from the article, e.g., when results were presented using population PK modeling; AUC = area under the concentration-time curve (μg/mL·h, μg/mL·min, μg/L·h or μmol/L·h); Cmax = maximum plasma concentration (μg/mL or μg/L); CL = clearance (L/h, L/min, mL/min or L/h/m2); Vss = volume of distribution at steady-state conditions; CRC = colorectal cancer; D >0.1 μM = duration drug concentration above 0.1 μM (h); ECOG = Eastern Cooperative Oncology Group score; FDA = Food and Drug Administration; iv = intravenous; MC = mamma carcinoma; MRT = mean residence time (h); NSCLC = non-small cell lung cancer; PK = pharmacokinetics; po = per os; RCC = renal cell carcinoma; SD = standard deviation; T ½ = terminal half-life (h or min); Vd = volume of distribution (L); Vdss = volume of distribution at steady state (L, or L/m2); VMel = maximal elimination capacity (μmol/h); y = year.

#### 4.1.2. Paclitaxel

Paclitaxel is highly plasma protein bound, with a large apparent volume of distribution. It is extensively metabolized in the liver by CYP3A4 and, to a lesser extent, by CYP2C8, into inactive metabolites [34]. Paclitaxel showed a high inter- and intra-patient PK variability [35]. A retrospective small Japanese study (*n* = 14) showed no PK differences with increasing age [36]. Two prospective small studies (*n* = 13, and *n* = 16, respectively) supported this similar PK profile [37,38]. However, one of these studies lacked a control group [37]. On the contrary, in another study (*n* = 23), increasing age was inversely associated with paclitaxel elimination, showing a 50% lower clearance of unbound paclitaxel in patients aged ≥70 years [39]. These results were supported by four larger studies showing an altered PK profile in elderly patients compared to their younger peers [40,41,42,43]. However, additional analyses of data from both large studies by Joerger *et al.* (*n* = 168, *n* = 273, respectively) showed that this age effect was predominantly driven by the youngest age group, with no PK differences in patients over 70 years of age compared to their younger peers. It remains uncertain whether elderly patients treated with paclitaxel in clinical practice have a similar PK profile compared to their younger peers, although recent large studies imply a small impact of age on paclitaxel PK [40,41,42]. Given the high inter- and intra-patient variability in paclitaxel PK, it seems implausible that any potential age-related PK difference has relevance in routine clinical practice.

### 4.2. Anthracyclines

#### 4.2.1. Doxorubicin

Doxorubicin has a high volume of distribution with extensive tissue uptake, moderate plasma protein binding and is metabolized by CYP enzymes [44,45]. About 50% of the parent drug is excreted in the bile, while 30% of doxorubicin is excreted as conjugates. Five to 12% of the drug is excreted renally. Due to physiological changes in the elderly, a different PK profile may be expected. FDA’s SmpC reported no overall differences in safety and effectiveness in elderly patients (estimated *n* = 4600) compared to younger patients. Conversely, the SmpC also recommended to consider a lower dose or longer interval between cycles in elderly women with metastatic breast cancer, without providing support or references [45]. Relatively small studies (*n* = 14 and *n* = 27, respectively) found no influence of advanced age on the clearance of doxorubicin [46,47], although one of these studies noted a trend towards a lower volume of distribution in elderly patients [47]. On the contrary, Robert *et al.* reported a significant decrease in the distribution phase in two different study populations (*n* = 37 with breast cancer, lymphoma or other tumors and *n* = 26 with solid tumors, respectively) [48]. However, increasing age did not significantly influence total clearance in these study populations. A subsequent pooled analysis of four small clinical trials by Li *et al.* (*n* = 56) supported this significant age-dependent impact on the distribution phase of doxorubicin. This study showed that initial doxorubicin concentrations were higher in the elderly patient due to a slower distribution of the drug into peripheral compartments, most probably due to altered regional blood flow. Furthermore, Li *et al.* described a small, but significant inverse correlation between age and total doxorubicin clearance [49]. These findings were supported by a study including 65 breast cancer patients, showing a significant 9% decrease in doxorubicin clearance per 10-year increase in age [50]. Overall, it seems questionable that these possible PK differences have clinical relevance in routine clinical practice.

#### 4.2.2. Epirubicin

Epirubicin is extensively and rapidly metabolized in the liver and eliminated in the bile [51]. In one study, including 78 breast cancer patients, no relevant age-related differences in PK of epirubicin were found. However, the number of elderly patients included in this study was not provided [52]. In another PK study (*n* = 36), on the contrary, a significant influence of increasing age on epirubicin clearance was observed, although it should be noted that only one patient aged over 70 was included in this analysis [53]. This finding was supported by a study including 66 breast cancer patients, showing an increased epirubicin maximum serum concentration with increasing age [54]. Based on these results, more rigorous monitoring in elderly women was recommended by the FDA [55,56].

### 4.3. Alkylating Agents

#### 4.3.1. Cyclophosphamide

Cyclophosphamide is a prodrug with low protein binding (24%), which is activated in the liver, predominantly by CYP2B6, CYP2C9 and CYP3A4, and shows autoinduction after repeated administration [57]. Following intravenous administration, cyclophosphamide is primarily excreted as metabolites, while 10%–20% is excreted unchanged in the urine and 4% is excreted in the bile. The impact of liver dysfunction on cyclophosphamide PK remains unclear at present, with some reports showing no association, while others reported increased toxicity and even hepatic failure [57,58,59]. Overall, insufficient data are available to provide specific advice for the elderly patient, although cautious dose selection is advised in this patient group [59]. One small and one moderate-sized study (*n* = 14, *n* = 65, respectively) showed no apparent impact of patient age on the clearance of cyclophosphamide [47,50].

### 4.4. Vinca-Alkaloids

#### 4.4.1. Vinorelbine (Intravenous)

Vinorelbine shows plasma protein binding between 60% and 91% and is extensively metabolized by the liver via CYP3A, with a renal clearance of only 11% [60]. Several small to moderate-sized clinical trials showed no correlation between age and elimination of vinorelbine [61,62,63,64,65]. Gauvin and colleagues, however, described a significant age-related decrease of vinorelbine clearance in two clinical studies that exclusively included patients over the age of 66 years, thus lacking a control group of younger patients. As acknowledged by the authors, the age-associated difference in clearance was mainly driven by the age group of 66–70 years [66,67]. Furthermore, differences in assay sensitivity, matrices used for PK analysis, divergent sampling times and duration and different patient populations may explain differences in PK parameters in these studies. Overall, available data do not support individual dosing based on patient age, as acknowledged by the drug’s SmPC [68].

#### 4.4.2. Vinorelbine (Oral)

Oral administration of vinorelbine may be an attractive alternative to intravenous vinorelbine for elderly patients, who are often affected by poor venous access. However, oral vinorelbine has been associated with severe and potentially fatal neutropenia, which was associated with high drug exposure [69]. Subsequently, an intraindividual dose escalation regimen was developed, which has received market access in several European and Asian countries and Australia, but has not received market approval in the USA [70,71,72]. The average bioavailability of the oral formulation of vinorelbine ranges from 33%–43%, with linear elimination similar to the intravenous formulation [63,64,73,74]. Several phase I/II studies showed no significant difference in PK parameters between the elderly population and their younger counterparts. Although some studies used historical data as a control, all results from separate study analyses and pooled data analyses suggested that increasing age has no clinically-relevant impact on vinorelbine PK [63,74,75].

### 4.5. Anti-Metabolites

#### 4.5.1. 5-Fluorouracil

After intravenous administration, 5-fluorouracil (5-FU) shows low protein binding and has a high apparent volume of distribution [76]. It is predominantly metabolized in the liver, where the enzyme dihydropyrimidine dehydrogenase (DPD) plays a crucial role in the deactivation of 5-FU [77,78]. After intravenous administration, approximately 90% of the dose of 5-FU is undergoing metabolic inactivation via DPD, and a small percentage undergoes renal excretion. In one study, a significant influence of aging on the AUC of 5-FU was documented, although the authors noted that the effect was weak with a remaining unexplained variation in AUC of more than 60% [79]. Another small study showed a non-significant trend towards lower clearance in the elderly patient [80]. These results were countered by three moderate to large-sized studies (*n* = 380, *n* = 103 and *n* = 31, resp.) showing no PK differences in the elderly population [81,82,83]. These data supported the SmPC’s statement that specific dosing recommendations solely based on a patient’s age are not necessary [84].

#### 4.5.2. Capecitabine

After oral administration, capecitabine is sequentially converted to 5′-deoxy-5-fluorocytidine (DFCR) by hepatic carboxylesterase and to 5′-deoxy-5-fluorouridine (DFUR) by CDA. The intermediate DFUR is converted to 5-FU by the enzyme thymidine phosphorylase in the final activating step. This protein has higher activity in tumor than in normal tissue. Although oral administration may be preferable in the elderly population, there is conflicting data as to whether elderly patients have a higher risk of developing severe toxicity with capecitabine compared to intravenous 5-FU [85,86]. Renal function and comorbidity were identified as independent predictors of dose reductions in elderly patients with colon cancer [87]. Twelves *et al.* showed that patients aged ≥70 years received significantly more dose reductions than their younger peers (51% *vs.* 39%) during capecitabine treatment [88]. Although not formally advised, several studies proposed a lower starting dose of 1000 mg/m^2^ twice daily instead of 1250 mg/m^2^ BID for elderly patients [87,89,90]. However, the optimal dose for elderly patients remains controversial, with conflicting study results and inadequate efficacy data to support lower starting doses [91,92,93,94]. Additionally, these trials were designed to evaluate toxicity profiles in elderly patients, and no PK data were available [85,86,87,88,89,90,94]. In FDA’s medical review at approval, a meta-analysis of phase I studies (*n* = 85, range 33–77 years) was performed, showing an increase in AUC and Cmax of α-fluoro-β-alanine (FBAL), the major renally-excreted metabolite, in elderly patients compared to their younger peers. This difference in FBAL PK was ascribed to altered renal function rather than aging [95]. In a pooled data analysis conducted by the FDA in 505 patients, a 15% increase in AUC of FBAL was described per 20% increase in age. However, no influence of age on the PK of 5-FU or other metabolites has been shown [96]. Although the impact of FBAL plasma concentrations on clinical toxicity remains unclear, clinically-relevant effects are not expected [97,98,99,100,101]. In one moderate-sized PK study, a lower capecitabine clearance in elderly patients was found (*n* = 29), which the authors attributed to the overall number of women included. As no differences in PK of the metabolites 5′-deoxy-5-fluorocytidine, 5′-deoxy-5-fluorouridine (DFUR) and the active 5-FU were reported, no dose adjustments were recommended for elderly patients [102]. Another moderate-sized study (*n* = 25) showed no influence of age, gender, BSA or creatinine clearance on PK parameters [98]. This was supported by a population PK analysis study including 20 elderly patients, compared to PK data of 40 younger patients from two previous clinical trials, showing no effect of age on the clearance of capecitabine and its metabolites. Although a difference in absorption by age was documented, the authors reported this as not having clinical relevance [101]. According to the drug’s SmPC, capecitabine should be used cautiously in elderly patients. EMA stated that no adjustment of the starting dose is needed, whereas according to the FDA, there is insufficient data to recommend specific dose adaptations for older patients [96,103]. Analysis at approval was in line with subsequent studies showing no clinically-relevant effect of aging on capecitabine PK. Still, elderly patients develop more pronounced toxicity from capecitabine on average, potentially due to reduced organ function, and a reduced initial dosing of the drug may be justified accordingly.

### 4.6. Platinum Agents

#### 4.6.1. Carboplatin

Carboplatin binds irreversibly to plasma proteins. The drug mainly undergoes renal excretion [104]. In clinical practice, carboplatin is dosed based on estimates of GFR, typically by using the Calvert formula. This results in a more predictable individual drug exposure as assessed by the area under the plasma concentration curve (AUC) and minimizes the risk of toxicity, especially in elderly patients with renal dysfunction. However, there remains some controversy about which formula may best predict target AUC in elderly patients [105,106]. In a phase I study including 55 patients receiving carboplatin dosed in mg/m^2^, a significant increase in mean plasma ultrafiltrate AUC of carboplatin was observed with increasing age [43]. In a more recent study (*n* = 24), elderly patients received carboplatin AUC 4 (mg/min/Ml) *versus* carboplatin AUC 5 in their younger counterparts, as calculated with the Calvert–Cockcroft–Gault formula. Despite this per protocol dose adjustment, exposure was similar in both groups. Age was a significant predictor of carboplatin clearance, showing a 31% mean clearance decrease in patients aged 70 years or over compared to younger patients [107].

#### 4.6.2. Cisplatin

Cisplatin binds rapidly and irreversible to proteins. Free platinum is mainly eliminated renally [108,109]. Cisplatin is formally contraindicated in patients with a measured or calculated GFR <60 mL/min [110,111,112], although dose adjustments may be applied with decreased renal function in clinical practice. In a moderate-sized PK study (*n* = 22), lower creatinine clearance was significantly associated with cisplatin maximum plasma concentrations (Cmax) [113]. Although the influence of age on cisplatin PK was not tested in this analysis, results could justify dose reductions or prolonged infusion of cisplatin in elderly patients with an associated decreases in renal function. In a single PK study (*n* = 23) including patients with normal renal function, increasing age was a significant predictor of lower clearance of total and free fraction platinum [114]. It should be mentioned, however, that cisplatin-associated nephrotoxicity was not increased in elderly patients, whereas increased myelosuppression was seen in some elderly patients receiving cisplatin, making arbitrary dose reductions based solely on aging disputable [111,115,116,117].

### 4.7. Monoclonal Antibodies

#### 4.7.1. Trastuzumab

Trastuzumab is a monoclonal antibody directed against the extracellular HER-2 receptor that is overexpressed in roughly 25% of breast cancer patients. After intravenous administration, trastuzumab undergoes target-directed distribution and elimination by the reticuloendothelial system and tissue turnover, with a long terminal half-life of 3–4 weeks. Trastuzumab is approved in patients with HER2-positive breast cancer in the early or advanced stage. While trastuzumab is given as a three-weekly intravenous infusion in early-stage breast cancer [118], it is usually given as a weekly infusion in patients with metastatic breast cancer [119]. In a large pooled dataset of 164 patients with early-stage and 76 patients with metastatic breast cancer, the authors performed a population PK-PD analysis to assess trastuzumab-associated cardiotoxicity [120]. While patient age was found to be a significant covariate on the relationship between trastuzumab PK and the trastuzumab-associated decrease of left ventricular ejection fraction (LVEF), the impact of patient age was considered marginal. Given the fact that trastuzumab-associated cardiotoxicity is the clinically most important adverse event, the analysis by van Hasselt and colleagues strongly argues against age-dependent dosing of trastuzumab. Monitoring of cardiac function at baseline and during trastuzumab treatment, however, is of substantial importance to keep a favorable benefit-risk profile also in the elderly breast cancer patient. This is also in agreement with data as described for the trastuzumab antibody-drug conjugate T-DM1 (see below).

#### 4.7.2. Trastuzumab Emtansine (Antibody-Drug Conjugate)

Trastuzumab emtansine (T-DM1) is a novel antibody-drug conjugate, which is composed of trastuzumab coupled through a stable thioether linker to mertansine, an anti-microtubule cytotoxic agent [121]. T-DM1 received fast-track market approval in 2013 in the European Union and the United States based on one large pivotal trial [122,123]. T-DM1 undergoes proteolytic degradation similar to trastuzumab. Yet, drug clearance of T-DM1 was faster than trastuzumab clearance, which may be ascribed to the addition of the deconjugation clearance pathway of T-DM1 [124]. Population PK analysis of multiple phase I–III studies revealed no influence of aging on the PK of trastuzumab emtansine in large datasets [125,126]. This is in line with the official label stating that age has no clinically meaningful effect on trastuzumab emtansine PK [121].

### 4.8. Tubulin Inhibitors

#### 4.8.1. Eribulin Mesylate

Eribulin mesylate is a microtubule inhibitor with cytotoxic properties. No major metabolites were found in human plasma, and it is predominantly excreted unchanged in the feces. Renal excretion of eribulin mesylate was shown to be less than 10% [127]. Eribulin received FDA and EMA approval for the treatment of locally-advanced or metastatic breast cancer refractory to both anthracyclines and taxanes. According to the drug’s SmPC, no specific dose adjustment is recommended in elderly patients, based on a similar safety profile, except for an increased proportion of asthenia/fatigue in elderly patients [128,129]. In the available FDA review for approval, a separate PK analysis of elderly (≥65 years, *n* = 63) *versus* younger patients (<65 years, *n* = 206) was provided. This analysis showed that the median clearance of eribulin mesylate was not influenced by age [130].

### 4.9. Oral Targeted Anti-Cancer Agents

#### 4.9.1. Everolimus

Everolimus is an oral inhibitor of the mTOR pathway that is extensively metabolized in the liver and predominantly eliminated through the bile. It is a substrate for CYP3A4, as well as for PgP [131]. The FDA’s SmPC stated that the clearance of everolimus is not dependent on age [132]. Although this was not described in the pivotal trial itself [133], a thorough description was provided in the FDA’s drug approval review (*n* = 61) [134]. Furthermore, a study performed by Xu and colleagues supported the lack of association between patient age and the PK of everolimus. Since this is not reported in the results section, but merely noted in the discussion, it is likely that this comment refers to FDA’s drug SmPC [135].

#### 4.9.2. Lapatinib

Lapatinib is a HER2 and EGFR targeted kinase inhibitor. It shows a variable bioavailability due to its low solubility and first-pass metabolism by CYP3A4/5. After gastrointestinal absorption, lapatinib is highly protein bound (99%) to albumin and alpha-1-acid-glycoprotein. Elimination is predominantly mediated by CYP3A4/5, whereas renal elimination accounted for less than 2% [136]. As stated by the FDA and EMA, there are no dosing recommendations provided for elderly patients as no data are available on the age-related PK of lapatinib [137,138,139,140]. Although no influence of higher age on the PK was described for several other -nibs [141], “absence of evidence is not evidence of absence” of any association between patient age and lapatinib PK, as no studies were available investigating the influence of aging on lapatinib PK [138].

## 5. Discussion

This article summarizes existing evidence on the impact of patient age on the PK of anticancer drugs used for the treatment of breast cancer (excluding endocrine drugs). Although PK results published on many anticancer drugs are conflicting, there is reasonable evidence for patient age to impact the PK of taxanes, anthracyclines, vinorelbine, capecitabine and platinum compounds. On the other side, potential PK differences in the aging population did not appear to be clinically relevant in the case of docetaxel, doxorubicin, vinorelbine and capecitabine. Additional analysis of paclitaxel also implies no clinically-relevant PK differences in the elderly. Furthermore, there is no indication that age influences the PK of cyclophosphamide, although data are limited. Even though no age-related PK differences were observed with several oral tyrosine kinase inhibitors [141], no PK analyses were conducted in elderly patients using lapatinib; hence, a potential difference cannot be ruled out.

Recent market approval of several newer anticancer drugs was accompanied by comprehensive population PK analyses to evaluate the influence of various covariates on the drug’s PK, including patient age. For eribulin mesylate, trastuzumab emtansine and everolimus, these model-based analyses showed no age-associated differences in drug PK, suggesting that the approved dosing regimen can be applied in elderly patients. It should be borne in mind, however, that these results were based on elderly patients who fulfilled the eligibility criteria of the respective clinical trial, thus including a homogeneous patient group with a good performance status. Due to the inclusion and exclusion criteria, these results cannot plainly be extrapolated to the elderly patient population in daily practice. Another important issue is the threshold beyond which changes in PK parameters are suggested to be clinically relevant. While no guidelines on handling and interpreting PK parameters with regards to clinical relevance have been published to our knowledge, some data have been published on the interpretation of covariate models in oncology, and a quantitative impact of at least 20% has been suggested for respective covariates on major PK parameters, mainly drug elimination, to suggest clinical relevance [142]. Finally, there is still the need to individually interpret the respective data for potential clinical relevance, and the use of data simulations may be one strategy for supporting data interpretation.

By design, different cut-off values were used in the literature to define the “elderly population”, using a threshold of 65, 70 or 75 years, respectively. Furthermore, many published studies were not powered to analyze age-related effects on anticancer drug PK. In the available elderly trials, the need for adequate PK-PD research in the heterogeneous elderly population was acknowledged, which is reflected by a lack of specific recommendations per anticancer drug for elderly women in the current breast cancer guidelines [143,144,145,146,147]. Some published guidelines have completely abstained from mentioning the elderly population [144,145,146]. The guidelines of the American Association of Clinical Oncology (ASCO) on advanced HER2-positive breast cancer however support the need for high quality data in elderly patients, yet omit providing specific treatment advice for the elderly population [143]. The respective European breast cancer guidelines state that treatment decisions should not be age dependent. The authors acknowledge that, although controversial, dose reductions and treatment regimen modifications are increasingly applied with increasing age. While combination chemotherapy is clearly more effective in the adjuvant breast cancer setting, single-agent sequential chemotherapy is advised to be the preferred option for elderly women with metastatic disease [147]. Growing emphasis on the influence of increasing age on treatment choice and outcome is discernible in several recent guidelines [143,147,148]. However, a paucity of adequate evidence-based data and conflicting trial results deters conclusive treatment strategies for the heterogeneous group of elderly patients treated in routine clinical practice. If substantial dose adaptations or dose reductions are considered in an elderly breast cancer patient, clinicians should consider the following issues: (1) potential organ dysfunction as a clear argument for impaired drug clearance; (2) extensive pretreatment with ensuing impairment of bone marrow reserve; (3) looking at objective geriatric scales, such as the Comprehensive Geriatric Assessment (CGA) or the Geriatric Prognostic Index (GPI) [149,150,151,152] to quantitate potential impairment of the general performance status; (4) checking for potential drug-drug interactions and polypharmacy; (5) a clinical pharmacologist to discuss with patients, if possible.

The PK profile of an anticancer drug in the elderly is not the sole predictor of treatment tolerability, as pharmacodynamic differences may also play an important role [153]. With docetaxel, for instance, an increased incidence of hematologic toxicity in the elderly [25,27] may be ascribed to deprivation of bone marrow and/or a greater sensitivity of bone marrow function to this chemotherapeutic agent without PK alterations. Altogether, this complicates a well-balanced management of the optimal choice of treatment and its corresponding dosage in the large group of elderly patients in daily practice. Ideally, clinical dosing regimens should be tailored to the specific patient (group). In order to predict the best standard of care for the heterogeneous group of elderly patients, it is important to clarify which factors influence the PK profile of anticancer drugs and to what extent. Therefore, it is of importance to include a representative subpopulation of geriatric patients in clinical trials and observational studies. Promising approaches to improve adequate PK-PD data acquisition in geriatric patients include: (1) pharmacometric modelling and clinical trial simulation techniques; (2) specific comorbidity and drug interaction studies; (3) optimal guidance and timely interaction with regulatory agencies; and (4) a holistic approach for the older cancer patient including international geriatric assessments, such as the Comprehensive Geriatric Assessment (CGA) or the Geriatric Prognostic Index (GPI) [150,151,152,153], and tailored study endpoints, including, e.g., impact on quality of life. Although some of these approaches are already being implemented in drug development programs and clinical practice, they should routinely be employed to achieve well-defined risk-benefit evaluations of treatment regimens used in the elderly population.

## 6. Conclusions

Selected published data revealed differences in the effect and magnitude of increasing age on PK of several anticancer drugs. There may be a clinically-relevant age-related PK difference for anthracyclines and platina agents. In the majority of cases, age is not a good surrogate marker for anticancer drug pharmacokinetics, and the physiological state of the individual patient may better be approached by looking at organ function, Charlson Comorbidity Score or geriatric functional assessment.
